# Preliminary Investigations Into the Effect of Exercise-Induced Muscle Damage on Systemic Extracellular Vesicle Release in Trained Younger and Older Men

**DOI:** 10.3389/fphys.2021.723931

**Published:** 2021-09-24

**Authors:** Yvoni Kyriakidou, Isabella Cooper, Igor Kraev, Sigrun Lange, Bradley T. Elliott

**Affiliations:** ^1^Translational Physiology Research Group, School of Life Sciences, University of Westminster, London, United Kingdom; ^2^Electron Microscopy Suite, Faculty of Science, Technology, Engineering and Mathematics, Open University, Milton Keynes, United Kingdom; ^3^Tissue Architecture and Regeneration Research Group, School of Life Sciences, University of Westminster, London, United Kingdom

**Keywords:** eccentric exercise, muscle damage, inflammation, extracellular vesicles, ageing, strength, delayed onset muscle soreness, recovery

## Abstract

**Background:** Exercise-induced muscle damage (EIMD) results in transient muscle inflammation, strength loss, and muscle soreness and may cause subsequent exercise avoidance. Research has recently proven that skeletal muscle can also release extracellular vesicles (EVs) into the circulation following a bout of exercise. However, EV’s potential role, including as a biomarker, in the response to eccentric resistance exercise stimulus remains unclear.

**Methods:** Twelve (younger, *n*=7, 27.0±1.5years and older, *n*=5, 63.0±1.0years) healthy, physically active males, undertaking moderate, regular physical activity (3–5 times per week) performed a unilateral high intensity eccentric exercise protocol. Venous plasma was collected for assessment of EVs and creatine kinase (CK) prior to EIMD, immediately after EIMD, and 1–72h post-EIMD, and maximal voluntary isometric contraction (MVIC) and delayed onset muscle soreness (DOMS) were assessed at all time points, except 1 and 2h post-EIMD.

**Results:** A significant effect of both time (*p*=0.005) and group (*p*<0.001) was noted for MVIC, with younger participants’ MVIC being higher throughout. Whilst a significant increase was observed in DOMS in the younger group (*p*=0.014) and in the older group (*p*=0.034) following EIMD, no significant differences were observed between groups. CK was not different between age groups but was altered following the EIMD (main effect of time *p*=0.026), with increased CK seen immediately post-, at 1 and 2h post-EIMD. EV count tended to be lower in older participants at rest, relative to younger participants (*p*=0.056), whilst EV modal size did not differ between younger and older participants pre-EIMD. EIMD did not substantially alter EV modal size or EV count in younger or older participants; however, the alteration in EV concentration (ΔCount) and EV modal size (ΔMode) between post-EIMD and pre-EIMD negatively associated with CK activity. No significant associations were noted between MVIC or DOMS and either ΔCount or ΔMode of EVs at any time point.

**Conclusion:** These findings suggest that profile of EV release, immediately following exercise, may predict later CK release and play a role in the EIMD response. Exercise-induced EV release profiles may therefore serve as an indicator for subsequent muscle damage.

## Introduction

Resistance exercise has a myriad of beneficial effects that can offset negative physiological effects associated with ageing and is highly recommended as a strategy to minimise loss in muscle mass and function across the lifespan, and to improve quality of life (Little and Phillips, 2009; Bei et al., 2017; Bagheri et al., 2019). In older adults, eccentric resistance exercise interventions have been suggested due to high load and potentially greater anabolic response at a low energy cost (Gault and Willems, 2013; Lim, 2016; Franchi et al., 2017). It has also been proposed that eccentric exercise-induced muscle damage (EIMD) may be used to develop safer and more effective personalised training and recovery protocols (Givli, 2015). However, unaccustomed exercise, especially high load eccentric muscle contractions, is associated with temporary muscle damage, muscle pain, reductions in muscle force output, an avoidance of repeated loading and transient muscle inflammation (Jouris et al., 2011; Hyldahl and Hubal, 2014; Owens et al., 2019).

Whilst the characteristics of this eccentric type of EIMD have been well defined in healthy young participants (Nosaka et al., 2002; Damas et al., 2016; Kyriakidou et al., 2021), less research has been conducted in older individuals. More specifically, ageing involves a reduction in function of most physiological systems, including muscle mass and function, and is coupled with increased inflammatory signalling (Franceschi and Campisi, 2014). Ageing has been also associated with decreased bone density, which in turn negatively affects physical performance (Reid et al., 2016). Better understanding of any mechanistic ageing-associated differences in muscle damage, inflammation, and pain responses may thus aid both our understanding of physiological differences in older individuals, and also ultimately aid personalised exercise prescription in this population.

Extracellular vesicles (EVs) are lipid-bilayer membrane vesicles, released from the cell of origin, are found in most body fluids and participate in cellular communication *via* transfer of cargo proteins and genetic material systemically between cells (Inal et al., 2013; Colombo et al., 2014; Lange et al., 2017; Turchinovich et al., 2019; Vagner et al., 2019). As EV cargo is comprised of a large range of proteins, enzymes, and genetic material, circulating EVs and their amount, composition, and profile reflect the physiological and pathophysiological condition. Therefore, EV profiles can be useful biomarkers and are easily isolated and quantified from a range of body fluids, including sera and plasma (Hessvik and Llorente, 2018; Ramirez et al., 2018).

Research into EV profiles has largely focussed on human pathologies, including cancer and autoimmune diseases (Withrow et al., 2016; Garcia-Contreras et al., 2017; Lange et al., 2017; Dolcetti et al., 2020; Urabe et al., 2020; Zhao et al., 2020), and is linked to crucial roles in the pathophysiology of inflammation-associated disorders, particularly in relation to larger sized EVs (Słomka et al., 2018). In comparison, explorations of the roles for EVs in normal physiology are fewer, with one plausible mechanism of action being adaptation and recovery from exercise stimuli. For instance, following treadmill running an increase in circulating EVs was seen in mice (Bei et al., 2017), whilst EV associated proteins were elevated in humans following 90min exhaustive aerobic exercise (Fruhbeis et al., 2015). Different intensities of aerobic treadmill exercise equally increased circulating EV concentrations, whilst increases in modal size were only seen with moderate intensity, not low or high intensity exercise (Oliveira et al., 2018). However, exercise modalities outside of endurance exercise have hitherto not been examined. Importantly, EVs have been shown to be involved in acute responses to injury and inflammatory stimuli in non-exercise models (Middel et al., 2016; Słomka et al., 2018). Whilst it is known that eccentric exercise induces the greatest magnitude of EIMD (Clarkson and Hubal, 2002; Herzog, 2014; Owens et al., 2019), it is likely that EVs will be involved in acute aspects of this response.

To our knowledge, no studies to date have examined the effect of eccentric exercise or the effect of ageing on circulating EV profiles following exercise. Therefore, this study aimed at isolating, quantifying, and size profiling EVs from the plasma of exercised human participants, to investigate whether there is any interplay between acute EIMD-induced changes in EV release profiles in younger and older participants, and whether such EV-related changes would correlate with other biological and muscle functional markers of EIMD, such as creatine kinase (CK) activity, strength, and muscle soreness.

## Materials and Methods

### Ethical Approval

Ethical approval was obtained by the College of Liberal of Arts and Sciences Research Ethics Committee, University of Westminster, United Kingdom (ETH1819-0328). All work herein conforms to the standards set by the Declaration of Helsinki. Written informed consent was obtained from all participants prior to their participation.

### Participants

Twelve (younger *n*=7, 27.0±1.5years and older *n*=5, 63.0±1.0years) healthy, physically active males, undertaking moderate, regular physical activity (3–5 times per week) volunteered to participate in this experimental study to perform a unilateral eccentric exercise protocol [seven sets of 10 repetitions at one repetition maximum (1RM), leg press machine]. The physical characteristics of the participants are presented in Table 1.

**Table 1:** Characteristics of participants at baseline.

	Total (*n*=12)	Younger (18–35; *n*=7)	Older (≥60; *n*=5)	*p*
Age (years)	42.00 (±5.32)	27.00 (±1.34)	63.00 (±0.93)	0.001^*^
Weight (kg)	74.02 (±2.77)	73.83 (±3.16)	74.30 (±5.46)	0.939
Height (cm)	180.50 (±1.45)	181.14 (±2.06)	179.60 (±2.16)	0.624
BMI (kg/m^2^)	22.73 (±0.77)	22.56 (±0.98)	22.98 (±1.35)	0.800
Body fat (%)	19.07 (±1.99)	16.90 (±2.60)	22.10 (±2.83)	0.212
Muscle mass (kg)	28.70 (±0.81)	29.53 (±1.04)	27.54 (±1.22)	0.243
1RM leg press (kg)	145.29 (±6.69)	152.16 (±7.66)	135.67 (±11.52)	0.241

Independent sample *t*-test comparison between younger (18–35years of age) and older (≥60years of age). Values are expressed as mean±SEM. BMI, body mass index; RM, repetition maximum.

* *p*<0.05.

Exclusion criteria included smoking, sex, taking any medication (e.g., non-steroidal anti-inflammatory drugs), and/or consuming anti-inflammatory (e.g., fish oil) supplements <6months prior to commencing the study and the presence of any known immune, cardiovascular or metabolic disease. To further confirm participants were free from upper respiratory tract infections, they completed an illness-specific questionnaire (WURSS-21; Barrett et al., 2009). Additionally, participants were free from any pain or injury as determined by the physical activity readiness questionnaire (PAR-Q) pre-exercise participation screening. Participants were also excluded if they regularly undertook downhill running or eccentric exercise (e.g., resistance exercise, squats, and lunges) as part of their normal training <6months prior to commencing the study. Participants were required to refrain from any exercise for 24h prior to baseline visit and 48h prior to EIMD visit, and from alcohol and caffeine 24h before baseline and EIMD visit. Further, they were asked to refrain from exercise during the recovery phase (for the subsequent 72h following the muscle-damaging exercise bout).

### Experimental Design

All participants were required to attend the human performance laboratory at the University of Westminster, London, United Kingdom, at the same time of day (±1h) in the morning on five occasions over a 2-week period. During visit 1 (baseline), in an overnight fasted-state, participants performed baseline measurements to ensure familiarisation of testing equipment and 5RM was determined. The baseline visit included anthropometric measurements, a venous blood sample, perceived muscle soreness, and maximal voluntary isometric contraction (MVIC) on the leg, described fully below.

**Figure 1:**
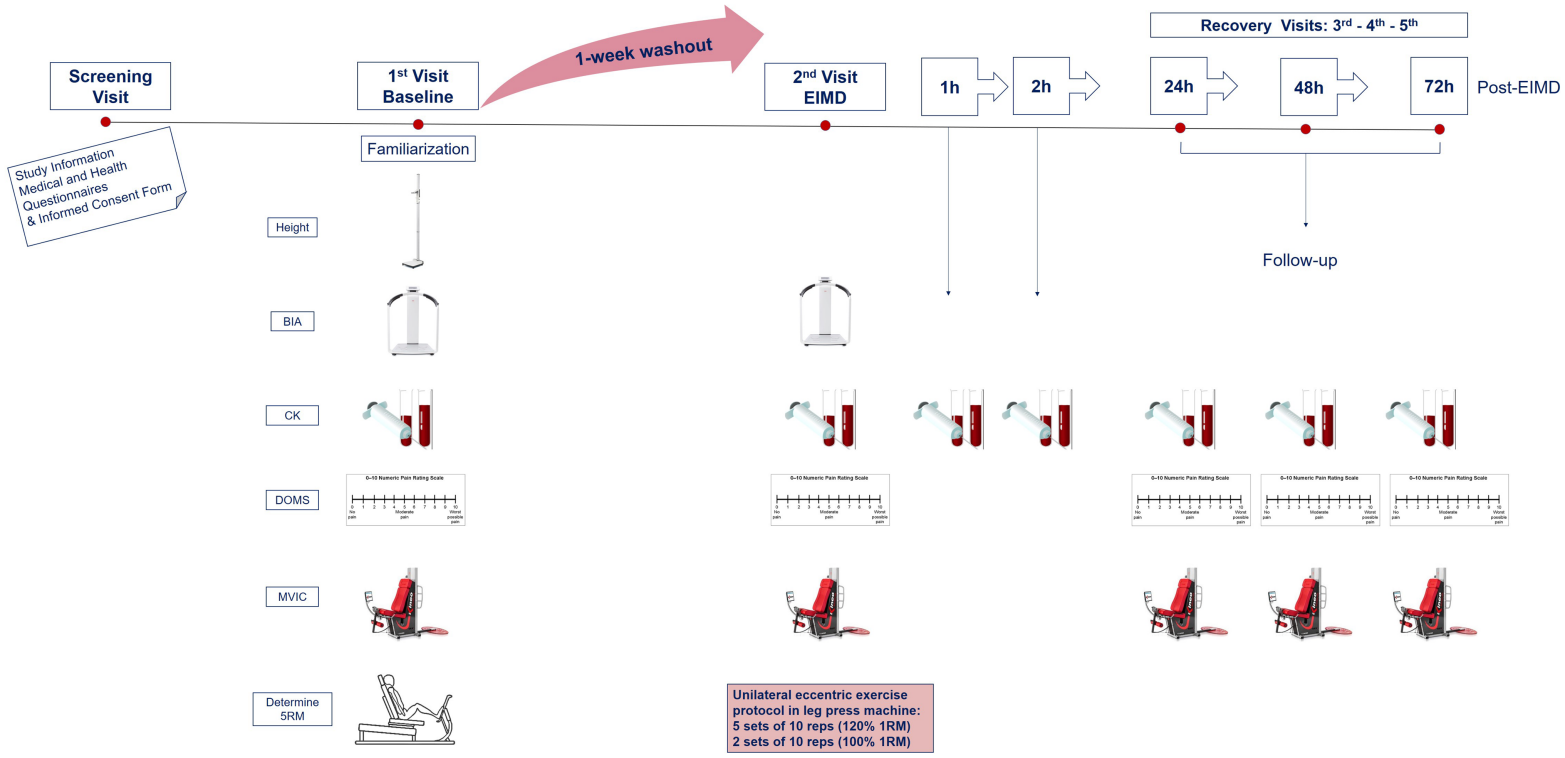
Schematic of experimental procedures. BIA, body impedance analysis; CK, creatine kinase; MVIC, maximal voluntary isometric contraction for peak force; DOMS, delayed onset muscle soreness *via* visual analogue scale; RM, repetition maximum; and EIMD, exercise-induced muscle damage. Second visit combines both pre- and post-measurements, immediately prior and following EIMD stimulus, respectively.

On visit 2 (7days later), participants reported to the laboratory at 07:00am having fasted overnight to complete the EIMD exercise protocol. All above measurements were repeated prior to (pre-EIMD) and immediately post (post-EIMD) the EIMD trial, and an additional blood sample was collected at 1 and 2h post-EIMD. Identical follow-up assessments were repeated at visits 3, 4, and 5 (24, 48, and 72h post-EIMD). An overview of the study design is presented in Figure 1.

### Anthropometric Measurements

Height (to nearest 0.1cm) was measured using a wall-mounted Holtain Harpenden Stadiometer (Holtain Ltd., Crymych, Wales, United Kingdom), and body weight (to nearest 0.1kg), BMI and body fat % (to nearest 0.1%) were measured using Seca® (mBCA 514 Medical Body Composition Analyzer, Gmbh&Co. KG, Hamburg, Germany) with participants being fasted, with an empty bladder and with standardised exercise clothing.

### Participants’ Determination of 1RM

A 5RM protocol was employed at baseline visit after anthropometric measurements, blood sampling and functional assessments to avoid residual EIMD from baseline affecting experimental measures. 5RM test was performed for the prediction of 1RM to minimise myofibrillar damage to the contractile proteins of the knee extensors, as well as to avoid adaptations to muscle damage and potential repeated bout effect for the EIMD trial. Each participant performed six concentric repetitions of incremental weight until failure, with 3min rest between sets. 5RM leg press predictive equation (Reynolds et al., 2006) was then applied to determine 1RM for each participant. The predicted 1RM weight lifted concentrically was then used to calculate 120% of the weight to be performed eccentrically at EIMD visit.

### Eccentric Leg Press Exercise Protocol

Participants performed a muscle-damaging exercise protocol known to successfully induce delayed onset muscle soreness (DOMS) in younger individuals (Vaile et al., 2008). The protocol comprised of seven sets of 10 eccentric single-leg press repetitions on a leg press machine (Body-Solid G9S Multi-Station Home Gym, Taiwan), with the first five sets of 10 repetitions at 120% of 1RM and final two sets of 10 repetitions at 100% of 1RM. A timed rest period of 3min took place between each set. The protocol was performed unilaterally on each participant’s dominant leg. Before performing each eccentric contraction, participants raised the weight using both legs, concentrically. Each eccentric contraction lasted 3–5s, during which participants resisted the load with the dominant leg from full knee extension to 90 degrees angle of knee flexion (Vaile et al., 2007, 2008). All participants completed all seven sets. Water was provided *ad libitum* every 15min.

### Assessment of Muscle Function

#### Muscle Soreness

Magnitude of DOMS was quantified using a visual analogue scale (VAS), and it was self-rated by participants on a 10-point-validated VAS indicating on a horizontal line with anchor points from 0 (no pain) to 10 (extreme pain; Carlsson, 1983; McCormack et al., 1988). Participants were seated with both legs in passive 90 degrees of flexion during a wall squat. Participants then placed a mark at the point on the VAS corresponding to their perception of soreness on the quadriceps muscle. Participants were blinded to the scores they had previously reported.

#### Maximal Voluntary Isometric Contraction

Maximal voluntary isometric contraction leg strength of the quadriceps was assessed on KINEO dynamometer (Globus Kineo 7000, Italy). Participants were seated upright and strapped into the dynamometer to limit excess motion. The chair was adjusted so that the leg pad was placed on the lower part of the tibialis anterior, and the pivot was located on the lateral epicondyle of the dominant leg. Maximal force was measured at an angle of 60 degrees leg extension. The protocol consisted of three maximal isometric contractions with 120s recovery between each repetition. Following a 2-min rest period, participants employed maximal isometric force against the leg pad. Peak force was determined by the average of three maximal isometric contractions lasting 3–5s. From pilot data (*n*=6 healthy younger participants) the within-day coefficient of variation (CV) for leg extension MVIC was calculated as 6.2% and the day-to-day CV was calculated as 8.7%. Verbal encouragement was given throughout each repetition.

### Venous Plasma

A 6ml vacutainer tube of venous blood was collected at each time point (lithium-heparin; BD, Oxford, United Kingdom). Whole blood was spun (Hettich Universal 320 R, Germany) at 3,857g for 10min at 4°C, with plasma aliquoted and frozen at −80°C.

Circulating CK activity was measured using a clinical chemistry analyser (Werfen ILab Aries, Italy). CK activity was determined using kinetic spectrophotometry at 340nm with a minimum detection limit of 3U/L, an undiluted linearity up to 900U/L. CV for CK was within run <1.2%, total <2.5%. All samples and standards were analysed in duplicate.

### EV Isolation and Characterisation From Human Plasma

#### Isolation of Plasma-EVs

Plasma EVs were prepared from the individual plasma (thawed on ice) aliquots (100μl per individual) from each participant, under the different conditions, using sequential centrifugation and ultracentrifugation according to previously standardised and described protocols and procedures (Kosgodage et al., 2018; Criscitiello et al., 2019; Pamenter et al., 2019), also following the recommendations of The International Society for Extracellular Vesicles (MISEV2018; Thery et al., 2018). For each individual plasma-EV preparation, 100μl of plasma was diluted 1:5 in Dulbecco’s PBS (DPBS, ultrafiltered using a 0.22μm filter, before use). This was then centrifuged for 20min at 3,000*g* at 4°C, to remove apoptotic bodies and aggregates. Supernatants were then collected and ultra-centrifuged at 100,000*g* at 4°C for 1h. This resulted in EV-enriched pellets, which were resuspended each in 500μl DPBS and thereafter ultra-centrifuged again for 1h at 100,000*g*, at 4°C. The final resulting EV pellets were resuspended each in 100μl of DPBS. The EV pellets were kept frozen at −80°C until used for nanoparticle tracking analysis (NTA) and transmission electron microscopy (TEM) in the procedures described below (all assessments were performed with EV preparations that had not been frozen for longer than 1week).

#### Nanoparticle Tracking Analysis

Plasma-EV quantification and size distribution profiles were established by NTA, based on Brownian motion of particles in suspension, using the NanoSight NS300 system (Malvern, United Kingdom). For NTA, the EV samples were diluted 1/100 in DPBS (10μl of EV preparation diluted in 990μl of DPBS). The diluted EV samples were applied to the NanoSight NS300 (Malvern Panalytical, United Kingdom), recording five repetitive reads, 60s each. Particle numbers per frame were 40–60, camera settings were at level 10 for recording and for post-analysis the detection threshold was set at 5. Replicate histograms were generated from these videos using the NanoSight software 3.0 (Malvern), representing mean and ±SEM of the five recordings for each sample.

#### Transmission Electron Microscopy

Plasma EVs were further assessed by morphological analysis using TEM. EVs were resuspended in 100mM sodium cacodylate buffer (pH 7.4). One drop (~3–5μl) of the EV suspension was placed onto a grid, which held a carbon support film which had been previously glow discharged. Following partial drying of the EV suspension, the sample was fixed for 1min at room temperature (RT) by placing the grid onto a drop of a fixative solution (2.5% glutaraldehyde) in 100mM sodium cacodylate buffer (pH 7.4). The grid was applied to the surface of three drops of distilled water for washing of the EV sample, removing excess water using a filter paper. The EVs were then stained for 1min with 2% aqueous uranyl acetate (Sigma-Aldrich), removing excess stain with a filter paper and air drying the grid. TEM imaging of EVs was carried out with a JEOL JEM 1400 transmission electron microscope (JEOL, Tokyo, Japan), which was operated at 80kV, using a magnification of 30,000x to 60,000x. Recording of digital images was performed with an AMT XR60 CCD camera (Deben, United Kingdom).

#### Western Blot Analysis

Extracellular vesicles were assessed for the EV-specific markers CD63 and Flotillin-1 (Flot-1), using western blotting. EV samples were diluted 1:1 in denaturing 2×Laemmli sample buffer (containing 5% beta-mercaptoethanol, BioRad, United Kingdom) and heated for 5min at 100°C. Protein separation was carried out at 165V using 4–20% gradient TGX gels (BioRad United Kingdom), followed by western blotting at 15V for 1h using a Trans-Blot® SD semi-dry transfer cell (BioRad, United Kingdom). Membranes were blocked with 5% bovine serum albumin (BSA, Sigma, United Kingdom) in Tris buffered saline (TBS) containing 0.1% Tween20 (BioRad, United Kingdom; TBS-T) for 1h at RT and primary antibody incubation was carried out overnight at 4°C using the EV-marker CD63 (ab216130, Abcam, United Kingdom) and Flot-1 (ab41927, Abcam); diluted 1/1,000 in TBS-T. The membranes were then washed at RT in TBS-T for 3×10min and thereafter incubated with HRP-conjugated anti-rabbit IgG secondary antibodies (BioRad), diluted 1/3,000 in TBS-T, for 1h at RT. The membranes were then washed for 4×10min TBS-T, and visualised, using enhanced chemiluminescence (ECL, Amersham, United Kingdom) in conjunction with the UVP BioDoc-ITTM System (Thermo Fisher Scientific, United Kingdom).

### Statistical Analysis

Normal distribution of data was examined by QQ plot visual inspection. Following Levene’s test of equality of variance, baseline characteristics were compared between groups using a two-tailed independent samples *t*-test. Exercise-induced changes in EV profiles, CK, and MVIC were analysed using a mixed model ANOVA with repeated measures [group (younger, older)×time (pre-, post-, at 1, 2, 24, 48, 72h post-EIMD)]. Tukey’s correction was used for *post hoc* analysis to perform pairwise comparisons. As an ordinal measure, Mann-Whitney U test was used to determine between group differences in DOMS. The EIMD effects on DOMS within-group were determined across time using Freidman ANOVA, and the Wilcoxon matched pairs signed ranks test was performed for *post hoc* analysis to test differences in this variable. The relationship between EV profiles, and CK, MVIC, and DOMS were performed with Pearson correlation. Partial eta-squared (η^2^_p_) values were calculated as measures of effect size for mixed model ANOVA when necessary, and were considered small (0.01), medium (0.06), or large (>0.14) effect, and for Wilcoxon matched pairs tests, effect size (*r*) was considered small (0.10), medium (0.30), or large (0.50) by the formula z/√n; where *n*=the number of observations over the two time points (Pallant, 2016); all were calculated using methods proposed by Cohen (1988). Values were considered statistically significant if *p*<0.05. Values were expressed as mean±SEM for data from parametric tests, and as median and interquartile range for data from non-parametric tests. All figures were generated in, and statistical analysis performed in GraphPad Prism (Version 9.1.1, GraphPad, United States), except generation of NTA curves which was carried out using the Nanosight 3.0 software (Malvern, United Kingdom). Subsequent power calculations on data presented within was calculated using G*Power (3.1.9.7).

## Results

Participant characteristics are presented in Table 1. Besides age, participants were reasonably homogenous, with no differences noted between body fat [younger 16.90 (±2.60) % vs. older 22.10 (±2.83) %, *p*=0.212], or muscle mass [younger 29.53 (±1.04) kg vs. older 27.54 (±1.22) kg, *p*=0.243].

**Figure 2:**
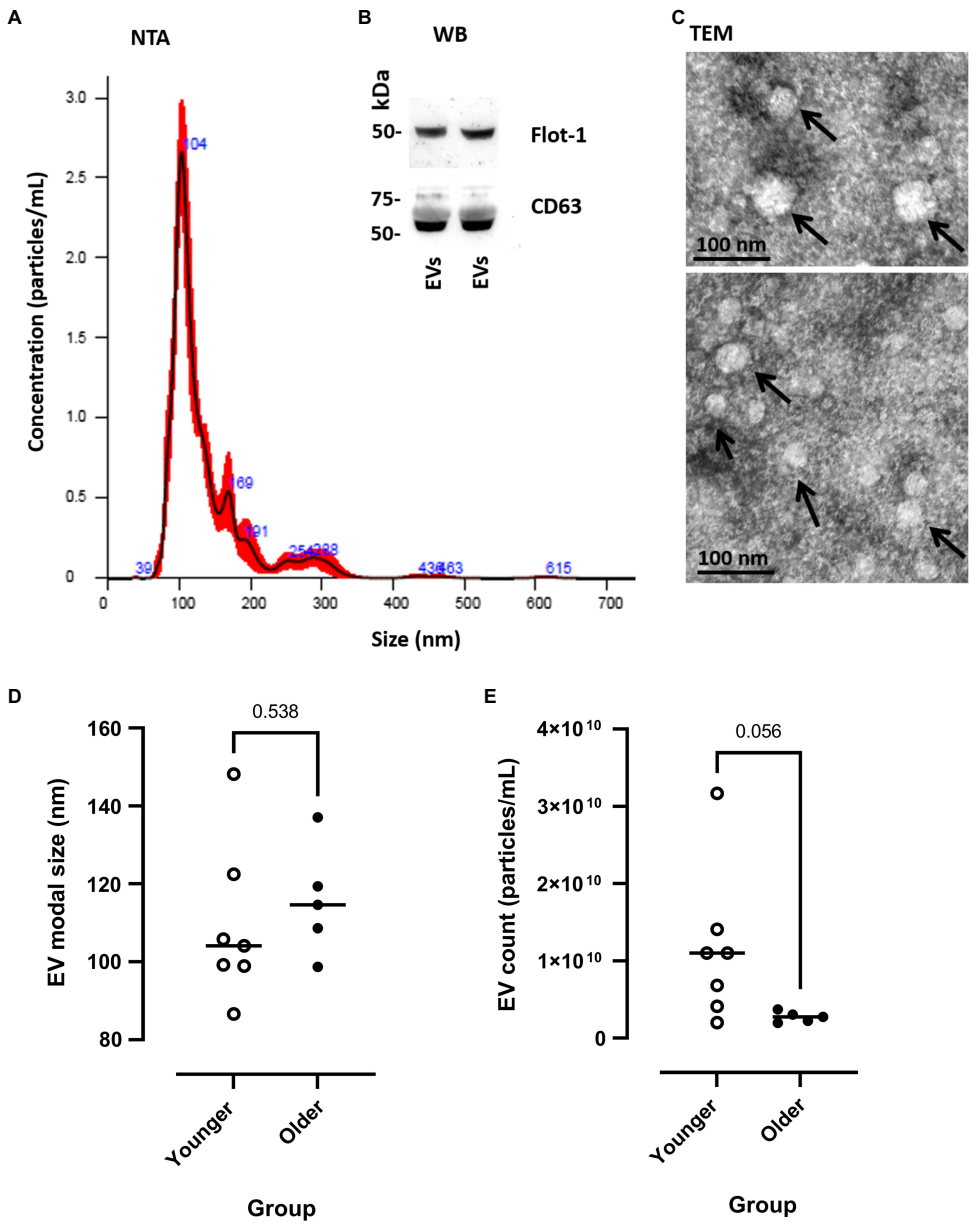
Measurement of EV modal size and count in younger and older participants. **(A)** Representative example of nanoparticle tracking analysis (NTA), SEM shown in red and mean in black line. **(B)** Western blotting of human plasma extracellular vesicles (EVs) showing positive for Flot-1 and CD63. **(C)** Transmission electron microscopy (TEM) images of human plasma-EVs, showing EV morphology; scale bar indicates 100nm. **(D)** EV modal size (nm) and **(E)** EV count (particles/ml) at pre-EIMD in younger (open circles) and older (closed circles) participants. Horizontal line indicates group means.

Extracellular vesicle profile (both modal size and particle concentration) was quantified by NTA (representative sample shown in Figure 2A) and were characterised by Western blotting for EV surface markers (CD63 and Flot-1; Figure 2B) and TEM for morphology (Figure 2C). Pre-exercise circulating blood samples suggested that EV modal size did not differ between younger and older participants [younger 109.33 (±7.64) vs. older 115.68 (±6.37) nm, *p*=0.538, Figure 2D], whilst EV count showed a trend of being lower in older participants at rest, relative to younger participants [younger 1.15×10^10^ (±3.72×10^9^) vs. older 2.75×10^10^ (±3.08×10^9^), *p*=0.056, Figure 2E].

**Figure 3:**
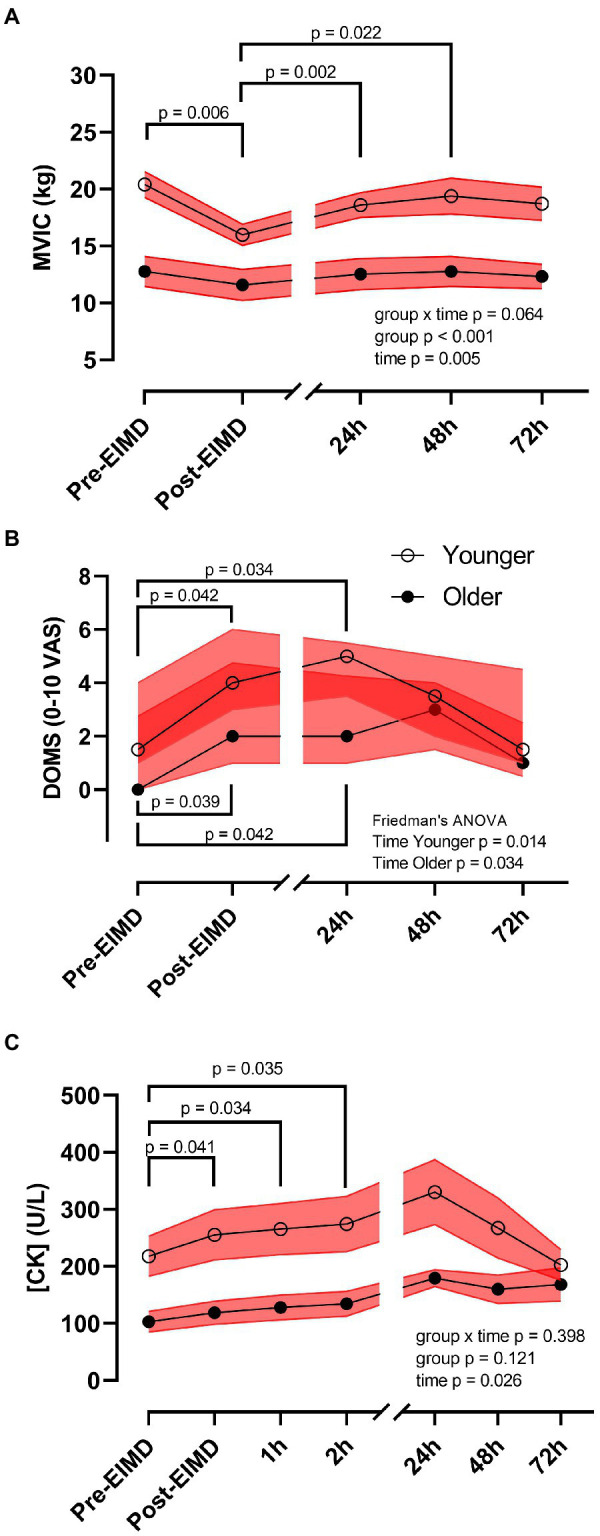
Exercise-induced muscle damage reduces muscle force and increases circulating creatine kinase (CK) concentrations independent of age. **(A)** MVIC (kg), **(B)** DOMS [0–10 visual analogue scale (VAS)], and **(C)** plasma CK (U/L) of younger (open circles) and older (closed circles) participants. Red shaded zones indicate SEM in **(A,C)**, and interquartile range in **(B)**, and black connected line indicates group means in **(A,C)**, and medians in **(B)**, values of *p* between timepoints as indicated. The scale brake indicates from hourly testing to 24-h intervals.

Repeated measures ANOVA showed no interaction between age group and time on leg MVIC (*p*=0.064). However, following the EIMD protocol, a main effect of both time (*p*=0.005, η^2^_p_=0.894) and age group (*p*<0.001, η^2^_p_=0.437) was noted for MVIC with a large effect size, suggesting that younger participants’ MVIC was higher throughout, and the EIMD protocol successfully reduced muscle force in both groups (Figure 3A). *Post hoc* testing suggests force significantly decreased immediately post-EIMD [pooled pre-MVIC, 17.21 (±1.40) kg to pooled post-MVIC, 14.15 (±0.99) kg, *p*=0.006], and then started to return in a linear recovery at 24h post-EIMD [pooled 24h MVIC, 16.07 (±1.22) kg, *p*=0.280] and at 48h post-EIMD [pooled 48h MVIC, 16.62 (±1.42) kg, *p*=0.845], but was not fully restored by 72h post-EIMD [pooled 72h MVIC, 16.05 (±1.32) kg, *p*=0.334].

Mann-Whitney U test showed no significant difference in DOMS between groups at any timepoint. However, following the EIMD protocol, Freidman test suggests a significant increase in DOMS across time in the younger group (*p*=0.014) and in the older group (*p*=0.034). Nevertheless, DOMS returned to pre-EIMD values by 72h post-EIMD in the younger group, but not in the older group (Figure 3B). *Post hoc* pairwise comparisons showed that both younger and older group had significantly elevated DOMS immediately post- [younger, Md=4.00 (3.00), *p*=0.042, r=0.54; older, Md=2.00 (3.75), *p*=0.039, *r*=0.65] and at 24h post-EIMD [younger, Md=5.00 (2.00), *p*=0.034, *r*=0.57; older, Md=2.00 (3.25), *p*=0.042, *r*=0.64] relative to pre-EIMD [younger, Md=1.50 (3.00) and older, Md=0.00 (2.75)], indicating a large effect size for both time points.

Exercise-induced muscle damage showed no group by time interaction on CK activity (*p*=0.398). However, CK was significantly altered following the EIMD (main effect of time *p*=0.026, η^2^_p_=0.519, suggesting a large effect size), with increased CK seen at immediately post-EIMD [pooled pre-CK, 170.18 (±27.26) vs. pooled post-CK 198.36 (±33.01) U/L; *p*=0.041], at 1h post- [pooled 1h CK, 208.26 (±33.78) U/L; *p*=0.034], and at 2h post- [pooled 2h CK, 216.13 (±35.40) U/L; *p*=0.035] EIMD completion. Circulating CK was not different between age group (Figure 3C, main effect of age group *p*=0.121).

**Figure 4:**
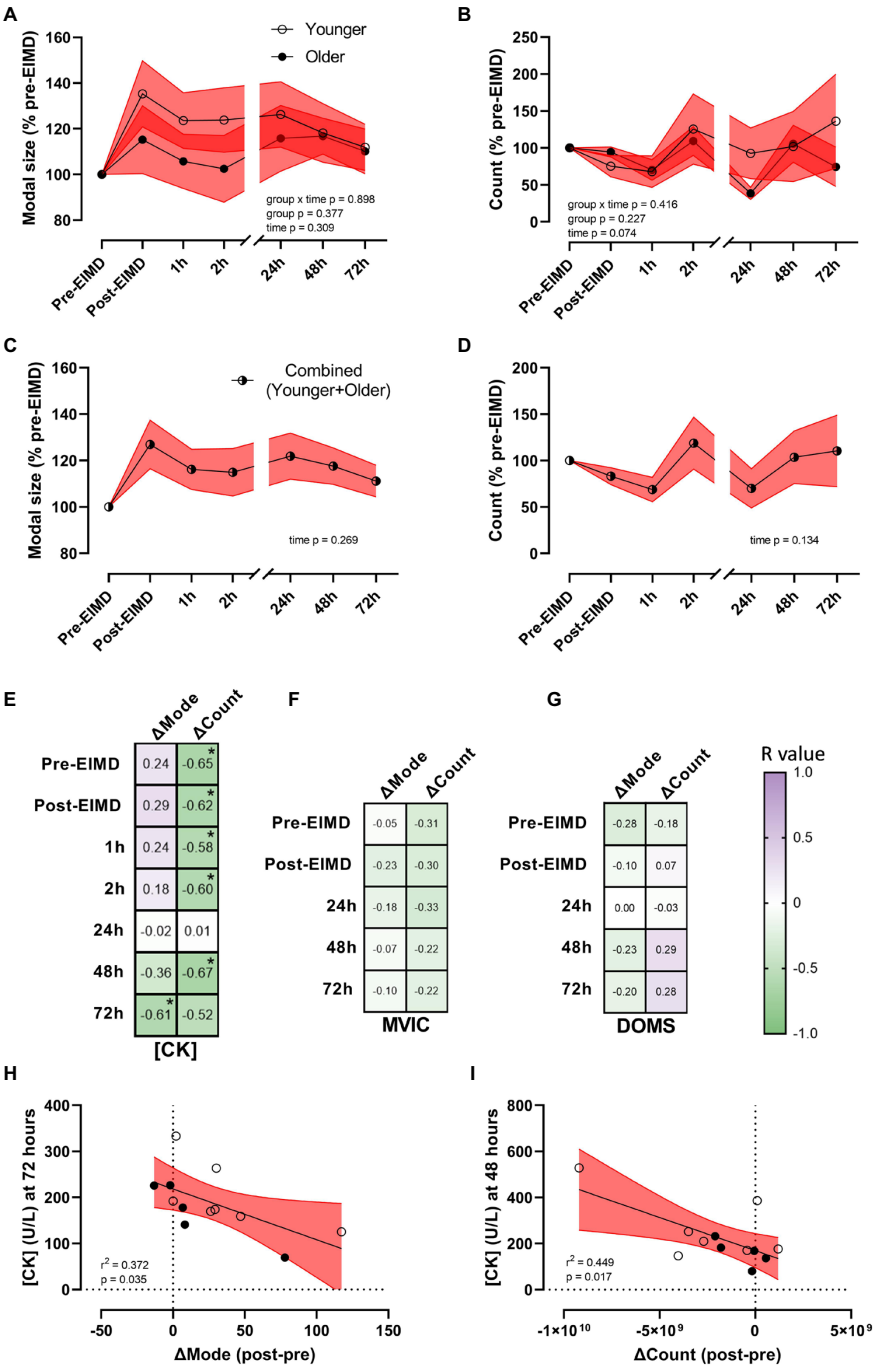
Alterations in EV modal size and count with exercise, and EV correlations with muscle damage markers. **(A)** EV modal size (% pre-EIMD) and **(B)** EV count (% pre-EIMD) as a function of timepoint between younger and older groups, **(C)** EV modal size (% pre-EIMD) and **(D)** EV count (% pre-EIMD) as a function of timepoint combined younger and older participants (*n*=12). Red shaded zones indicate SEM and black connected line indicates group means. The scale brake indicates from hourly testing to 24-h intervals. **(E)** Correlation matrix between change in EV modal size (ΔMode) or in EV count (ΔCount) as a function of CK (U/L), **(F)** as a function of MVIC (kg) and **(G)** as a function of DOMS (0–10 VAS) at each timepoint measured, with *r* values as shown. ^*^Indicates significant association between variables (each *p*<0.05). Colour intensity for *r* values (purple indicates positive *r* value, green negative, white=0) as indicated. **(H)** CK (U/L) at 72h as a function of ΔMode (post-EIMD – pre-EIMD and **I**) CK (U/L) at 48h as a function of ΔCount (post-EIMD – pre-EIMD). Red shaded zone indicates 95% CIs. Open circles indicate younger, closed indicate older.

Whilst the EIMD protocol visually appeared to induce increased expression and greater variability in circulating plasma-EV modal size in the younger group (Figure 4A), repeated measures ANOVA suggested EIMD had no significant effect on group by time interaction (*p*=0.898), nor a main effect of either group (younger or older, *p*=0.377), or time (*p*=0.309; Figure 4A). In a similar manner, the EIMD protocol did not substantially alter plasma-EV count, with no group by time interaction (*p*=0.416), nor a main effect of group (younger or older, *p*=0.227) or time (*p*=0.074; Figure 4B). These results are maintained if participants are examined independent of age (*n*=12), with one-way ANOVA suggesting no effect of time on EV modal size (*p*=0.269; Figure 4C) or count (*p*=0.134; Figure 4D). As a preliminary study into changes in EV profile with EIMD in younger and older participants, required sample size for future studies using a condition×time model with seven time points (as presented in Figure 4) was calculated as *n*=398 per group for EV modal size, and *n*=57 per group for EV count (*α*=0.05, power (1−*β*=0.8), effect size of 0.035 for EV modal and 0.093 for EV count).

To explore a correlation between EV release profiles as a putative biomarker of muscle damage, the numerical difference in EV modal size (ΔMode) and EV count (ΔCount) between post-EIMD and pre-EIMD was examined relative to CK (U/L), MVIC (kg), or DOMS at each time point measured. A significant association between ΔMode and circulating CK was seen at 72h only, post-EIMD (Figure 4E; *r*^2^=0.372, *p*=0.035 visualised in Figure 4H). Circulating CK was shown to significantly associate negatively with ΔCount at every time point measured, except 24h post-EIMD (Figure 4E; largest *r*^2^=0.449 at 48h visualised in Figure 4I, *p*=0.017). No significant associations were noted between MVIC and either ΔMode or ΔCount (Figure 4F), or DOMS and either ΔMode or ΔCount at any time point measured (Figure 4G).

## Discussion

Exercise is associated with a number of immediate physiological responses. Circulating EVs can act as plasma-based biomarkers, reflecting physiological and pathophysiological conditions of the body (Withrow et al., 2016; Zhao et al., 2020). Thus, this study analysed EVs in blood plasma isolated during the acute phase of EIMD and during a recovery period of 72h in younger and older healthy, physically active male adults. In this study, we show that a single bout of EIMD triggers apparent changes to EV concentration and size distribution profiles, but in trained older men there is no clear differences in this EV signature from that of younger men. However, unlike prior studies on the effects of acute endurance exercise on EV release profiles, acute eccentric resistance exercise does not appear to predictably alter EV modal size or EV concentration. Furthermore, immediate changes in EV profiles as observed here may associate with later changes in biological markers of muscle damage, such as CK, as found in the current study.

No significant effect on EV profiles was observed in relation to age at pre-exercise values, with younger and older participants showing relatively homogeneous EV profile responses. Nonetheless, older participants had lesser magnitude of CK response than their younger counterparts. Whilst the younger group showed a greater signal in CK response and returned to pre-exercise values by the end of the experimental period, suggesting a better resolution in recovery, the older group did not attain absolute values by the end of the recovery period. Unexpectedly, both groups had similar recovery in leg strength changes following EIMD. Likewise, a previous study has reported no age differences in muscle function after muscle-damaging exercise (Heckel et al., 2019). However, others concluded that younger individuals were able to recover and adapt quicker in functionality following EIMD, confirming that muscle function declines through the ageing process (Tieland et al., 2018; Fernandes et al., 2019). In the current study, muscle soreness significantly peaked immediately post- and at 24h post-EIMD for both groups, but the younger group consistently scored higher on perception of pain than the older group during the experimental period. This may have been attributed to a higher muscle damage, as indicated by the increased CK activity for the younger men, or hypothetically due to the larger ratio of type II fibres typically seen in younger individuals, which have been suggested to be more susceptible to injury (Byrne et al., 2004; Verdijk et al., 2014). However, muscle biopsies would be required to confirm the fibre type shift. Similarly, Lavender and Nosaka (2006) reported older males experienced lower muscle soreness than younger males following EIMD. A review by Gibson and Helme (2001) also reported that pain perception is decreased with ageing. This may explain the lower DOMS score of the older group compared with the younger group in the present study. Nevertheless, no significant differences were observed between groups following EIMD. Similarly, Nikolaidis (2017) and Heckel et al. (2019) demonstrated no differences between age groups after lower-body resistance exercise. However, Lavender and Nosaka (2008) found opposite findings after eccentric exercise. The contrast in research findings was attributed to the magnitude of muscle damage induced by the exercise protocol used (bilateral vs. unilateral) or due to the different muscle group (arm vs. leg) involved in the studies. Overall, the current study showed that EIMD recovery took a similar course in both muscle function and DOMS for physically active younger and older individuals. Therefore, the data presented here suggests that when younger and older individuals are matched for activity status, ageing does not appear to impair recovery from voluntary eccentric exercise.

Endurance exercise has been shown to alter EV profiles (Oliveira et al., 2020; Soares et al., 2021). Chronic exercise in murine models (3weeks swim training) was, for example, shown to significantly increase serum EV count (Bei et al., 2017), whilst the modal size of EVs was unchanged. Both EV count and modal EV size were elevated in race horses following a single bout sustained (160km) endurance exercise (Oliveira et al., 2020), which may correlate with previous observations of larger EVs being associated with inflammation (Słomka et al., 2018). Alternatively, in humans Fruhbeis et al. (2015) reported a significant increase in EV concentration immediately after an incremental cycling exercise to failure (typically 12–20min), but EVs were found to be cleared from the circulation during the early recovery period (90min after exercise). However, the concentration of plasma-EVs remained elevated after exhaustive running. In murine models, Oliveira et al. (2018) showed that 40min of moderate intensity endurance exercise immediately increased EV modal size, but neither low, nor high intensity exercise had any effect on modal size. It is therefore of interest that in the current study we did observe a shift in EV modal size towards larger EVs, at 48h post-EIMD (Supplementary Figure 1), albeit this trend was not statistically significant. Our findings are also in line with previous work of Lovett et al. (2018) who reported no significant change in EVs size or number over time after an acute muscle-damaging exercise (combination of plyometric jumping and downhill running). Thus, it may be that exercise duration, intensity, and modality, in addition to differential species responses may yield variable results, and this warrants further exploration to fully understand effects of an acute exercise bout on circulating EVs. Indeed, as already noted, great individual variability is observed in human responses to various exercise modalities, and thus differing EV profile response may in part underlie differing adaptation to these modalities (Trovato et al., 2019). Alternatively, in lieu of changes to the number and morphology of circulating EVs, their transported cargo may be more relevant to the exercise response, and thus future studies may choose to examine this variable.

Unlike research on endurance models, the current literature is lacking in resistance training investigations, and specifically eccentric muscle-damaging protocols, such as those used in the current study. Whilst Cui et al. (2017) assessed three different types of resistance exercise, they reported only changes in circulating microRNAs (miRNAs), not in EV profile states. Our study provides evidence that early changes in EV profile following EIMD significantly correlate with subsequent changes in CK, a known biomarker of muscle damage, and thus acute changes in EV profile post-exercise may indicate subsequent magnitude of muscle damage. Both processes may result from the mechanical EIMD stimulus (e.g., the mechanical “stretching” of the muscle cell membrane may promote both CK and EV release). Alternatively, it is tempting to speculate that the EV response may be causative of subsequent changes in muscle damage markers, such as CK; however, such causality is not possible to ascribe with the data collected here. Outside of EIMD, other types of muscle damage, such as laser membrane ablation and cellular hypoxia, have been reported to induce rapid increases in EV release; however, these have hitherto been performed on either zebrafish (Middel et al., 2016) or mouse models (Scheffer et al., 2015), muscle tissue *ex vivo*, and thus the results presented here are the first to extend these findings into human models of muscle damage.

Whilst associations have previously been observed between EV release profiles in response to inflammatory disorders (Hosseinkhani et al., 2018) and older individuals are noted to have elevated basal systemic inflammatory cytokines concentrations (Franceschi and Campisi, 2014), circulating miRNAs (Jung and Suh, 2014) and increased EV release is seen from senescent cells (Hitomi et al., 2020; Riquelme et al., 2020). Therefore, we were interested in examining any putative age differences in EV release profiles between older and younger individuals following a bout of EIMD. Whilst our results presented here suggest no major differences in EV modal size or EV plasma concentration in younger vs. older individuals, following a single bout of EIMD, some caution should be taken in the interpretation of these results due to the small sample size assessed and volunteer selection. The findings presented here are with recreationally active younger and older participants, all participants habitually engaged in structured physical activity, and thus are not representative of wider physically inactive Western populations (Farrell et al., 2013; Lindsay et al., 2019). Importantly also, in the ageing population, reduced physical activity and increase in sedentary time are typically observed (Lindsay et al., 2019). Furthermore, no difference in muscle mass or fat mass was seen in our study population, unlike that witnessed in wider society (Volpi et al., 2004; Barrios-Silva et al., 2018). By studying highly active ageing cohorts, we can separate physiological differences of ageing from inactivity induced changes (Harridge and Lazarus, 2017). Our results, therefore, should be interpreted in light of the relatively physical trained cohort presented here. Any potential differences suggested by the results presented here between age groups may be enhanced when expanding this study to exercise naive younger and older individuals; however, this may reflect effects of long-term inactivity, not ageing *per se*.

Whilst this pilot study on EIMD has presented some interesting results in relation to EVs as putative biomarkers for muscle damage, these findings will need further validation in larger cohorts that can be guided in sample size collection by the results presented here. Future investigations should also conduct in depth analysis of EV cargo composition will be of considerable interest for the identification of EV-related biomarkers in EIMD. Therefore, it will be of great interest to perform full EV profiling analysis using RNA sequencing, proteomics and metabolomics to reveal the EV cargo profiles in response to EIMD, also in different age populations. Whilst EV cargo biomarkers have been implicated in the pathophysiology of inflammation-associated disorders, research regarding their role in EIMD and ageing remains limited. This study therefore provides the first insights into the potential of EV-profiling in association with muscle-damaging exercise and ageing and paves the way for future studies, aiming to extend current knowledge on their roles as mediators of health-promoting effects, and as biomarkers, associated with physical activity.

In conclusion, here we show that physical responses to eccentric exercise induces plasma-EV changes that correlate with CK release post exercise, a biological marker of muscle damage. EV profiles did not appear to change significantly in relation to age groups assessed (active younger vs. older), which importantly may make them a reliable biomarker to assess effects of exercise interventions across age groups. As EV release has previously been associated with small animal models of muscle damage, our study further supports that EV release profiles immediately following exercise may also play a role in the EIMD response in humans. If the post-exercise EV response does indeed reflect physiological injury recovery responses, the magnitude and content of EV profile changes could be of interest for strategies to reduce the impairing effects of EIMD.

## Data Availability Statement

The raw data supporting the conclusions of this article will be made available by the authors, without undue reservation.

## Ethics Statement

The studies involving human participants were reviewed and approved by College of Liberal of Arts and Sciences Research Ethics Committee, University of Westminster, United Kingdom. The patients/participants provided their written informed consent to participate in this study.

## Author Contributions

YK and BE: conceptualisation, methodology, project administration, and writing – original draft preparation. YK, IK, and SL: formal analysis and investigation. YK: human fitness testing. YK, IC, IK, SL, and BE: data curation. YK, IK, SL, and BE: visualisation. BE: supervising and funding acquisition. YK, IC, SL, and BE: writing – review and editing. All authors contributed to the article and approved the submitted version.

## Funding

BE was supported by the Quintin Hogg Charitable Trust. This work was supported in part by publication funds provided by the British Society for Research on Ageing.

## Conflict of Interest

The authors declare that the research was conducted in the absence of any commercial or financial relationships that could be construed as a potential conflict of interest.

## Publisher’s Note

All claims expressed in this article are solely those of the authors and do not necessarily represent those of their affiliated organizations, or those of the publisher, the editors and the reviewers. Any product that may be evaluated in this article, or claim that may be made by its manufacturer, is not guaranteed or endorsed by the publisher.

## Figures and Tables

**Table:** 

Term	Definitions
ANOVA	Analysis of variance
BIA	Bioelectrical impedance analysis
BMI	Body mass index
BSA	Bovine serum albumin
CK	Creatine kinase
CV	Coefficient of variation
DOMS	Delayed-onset muscle soreness
ECL	Enhanced chemiluminescence
EIMD	Exercise-induced muscle damage
EVs	Extracellular vesicles
Flot-1	Flotillin-1
miRNAs	microRNAs
MVIC	Maximal voluntary isometric contraction
NTA	Nanoparticle tracking analysis
PAR-Q	Physical activity readiness questionnaire
Post-EIMD	Following exercise-induced muscle damage
Pre-EIMD	Before exercise-induced muscle damage
RM	Repetition maximum
RT	Room temperature
SEM	Standard error mean
TBS	Tris buffered saline
TEM	Transmission electron microscopy
VAS	Visual analogue scale
WURSS	Wisconsin upper respiratory symptom survey
ΔCount	Difference between EV concentration at post-EIMD and pre-EIMD time points
ΔMode	Difference between in EV modal size at post-EIMD and pre-EIMD time points

## Supplementary Material

Supplementary Figure 1 Representative example of EV profiles responses of a younger and older participant, showing shifts of EV modal size to larger EVs in response to EIMD during the post-exercise recovery period.
