# Supplementary figures and images for: Preliminary Investigations Into the Effect of Exercise-Induced Muscle Damage on Systemic Extracellular Vesicle Release in Trained Younger and Older Men
Source: Front Physiol. 2021 Sep 24;12:723931. doi: 10.3389/fphys.2021.723931 (PMC8507150; doi:10.3389/fphys.2021.723931)

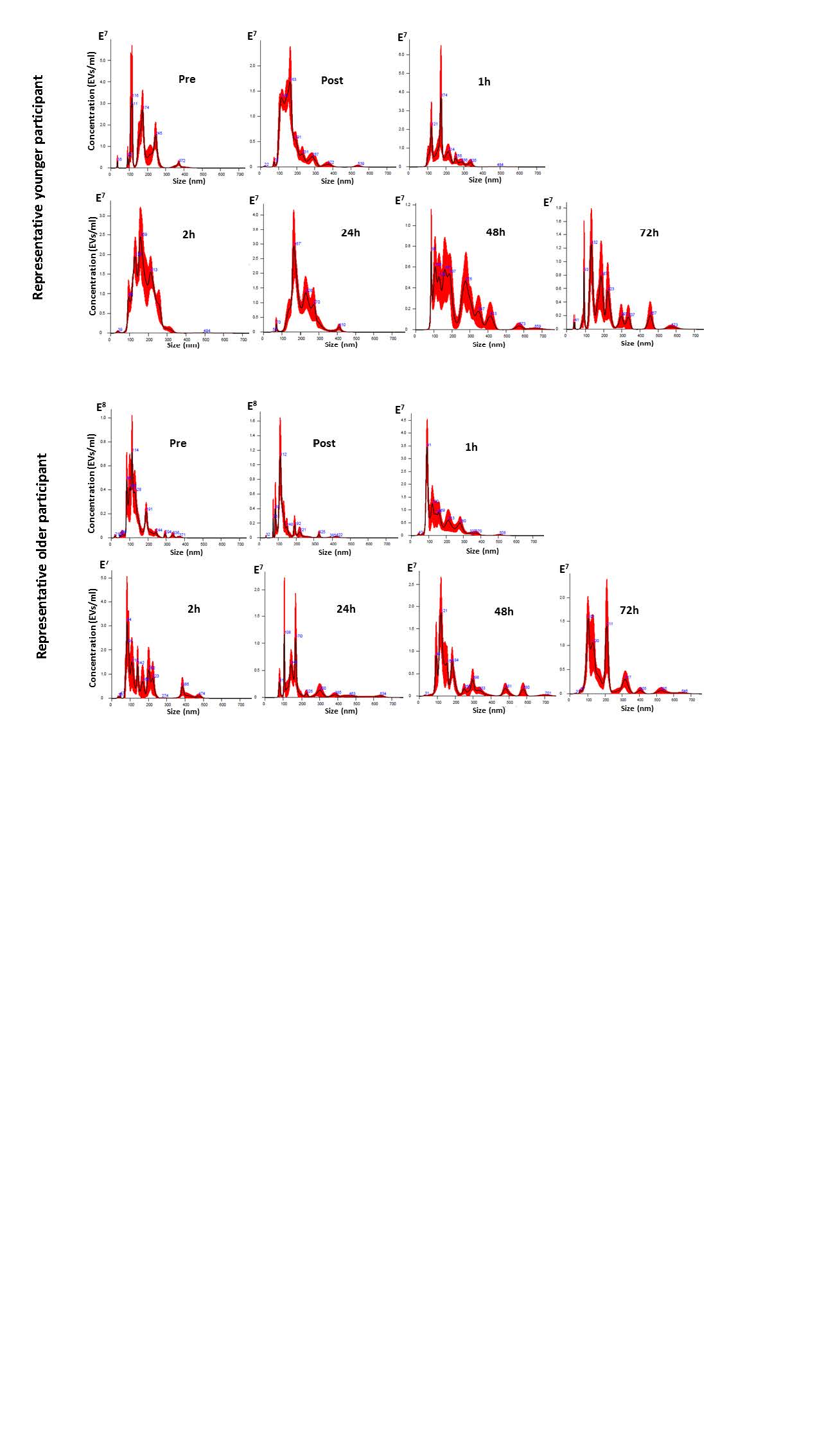

Supplement: Supplementary Figure 1 — Representative example of EV profiles responses of a younger and older participant, showing shifts of EV modal size to larger EVs in response to EIMD during the post-exercise recovery period. [file Image_1.JPEG]
